# The demographic, laboratory and genetic factors associated with long Covid-19 syndrome: a case–control study

**DOI:** 10.1007/s10238-023-01256-1

**Published:** 2024-01-17

**Authors:** Ensiye Torki, Fahimeh Hoseininasab, Marjan Moradi, Ramin Sami, Mark J. M. Sullman, Hamed Fouladseresht

**Affiliations:** 1 Department of Immunology, School of Medicine, Isfahan University of Medical Sciences, Isfahan, Iran; 2 Department of Genetics, School of Science, Shahrekord University, Shahrekord, Iran; 3 Department of Internal Medicine, School of Medicine, Isfahan University of Medical Sciences, Isfahan, Iran; 4 Department of Life and Health Sciences, University of Nicosia, Nicosia, Cyprus; 5 Department of Social Sciences, University of Nicosia, Nicosia, Cyprus

**Keywords:** Long Covid-19 syndrome, Severity, Interleukin-6, HLA class I

## Abstract

Long Covid-19 syndrome (LCS) manifests with a wide range of clinical symptoms, yet the factors associated with LCS remain poorly understood. The current study aimed to investigate the relationships that demographic characteristics, clinical history, laboratory indicators, and the frequency of HLA-I alleles have with the likelihood of developing LCS. We extracted the demographic characteristics and clinical histories from the medical records of 88 LCS cases (LCS^+^ group) and 96 individuals without LCS (LCS^−^ group). Furthermore, we evaluated the clinical symptoms, serum levels of interleukin (IL)-6 and tumor necrosis factor-α, laboratory parameters, and the frequencies of HLA-I alleles. Following this we used multiple logistic regression to investigate the association these variables had with LCS. Subjects in the LCS^+^ group were more likely to have experienced severe Covid-19 symptoms and had higher body mass index (BMI), white blood cell, lymphocyte counts, C-reactive protein (CRP), and IL-6 levels than those in the LCS^−^ group (for all: *P* < 0.05). Moreover, the frequencies of the HLA-A*11, -B*14, -B*38, -B*50, and -C*07 alleles were higher in the LCS^+^ group (for all: *P* < 0.05). After adjusting for the most important variables, the likelihood of suffering from LCS was significantly associated with BMI, CRP, IL-6, the HLA-A*11, and -C*07 alleles, as well as a positive history of severe Covid-19 (for all: *P* < 0.05). Our study showed that a history of severe Covid-19 during the acute phase of the disease, the HLA-A*11, and -C*07 alleles, higher BMI, as well as elevated serum CRP and IL-6 levels, were all associated with an increased likelihood of LCS.

**Supplementary Information:**

The online version contains supplementary material available at 10.1007/s10238-023-01256-1.

## Introduction

In May 2020, the term “Long Covid-19 Syndrome (LCS)” was introduced in response to reports of persistent initial symptoms, the recurrence of previously resolved symptoms, or the appearance of new symptoms in some individuals who had recovered from the acute phase of severe acute respiratory syndrome coronavirus (SARS-CoV)-2 infection [1, 2]. Subsequently, the World Health Organization (WHO) established a clinical case definition to facilitate a consensus description of LCS. According to this definition, LCS is characterized by the continuation or emergence of new symptoms at least three months after the initial SARS-CoV-2 infection. These symptoms persist for at least two months and cannot be explained by any other diagnosis [3]. Global estimates indicate that 54% of hospitalized and 34% of non-hospitalized Covid-19 patients experience long-term symptoms following recovery from the acute phase of the disease [4]. The diverse range of symptoms in LCS, includes fatigue, dyspnea, cough, cognitive and mental disorders, headache, chest and joint pains, loss of smell and taste, hair loss, insomnia, as well as cardiovascular and gastrointestinal issues, suggesting that LCS affects multiple organs [5, 6]. However, despite recent advances, the pathogenesis of LCS remains poorly understood. Several hypotheses have been suggested regarding the pathogenic mechanisms of LCS, including persistent ineffective antiviral immune responses [7, 8], which may result in long-term symptoms caused by direct cytopathic effects, chronic inflammation, and autoimmunity [2].

Given the substantial number of individuals affected, the diverse array of symptoms and phenotypes, and the absence of treatment and rehabilitation guidelines, LSC has emerged as an important global health challenge. LCS not only reduces an individual’s ability to resume their daily lives and work, but also increases the healthcare burden and economic losses caused by the long-term complications of Covid-19 [9, 10]. These issues highlight the need to identify diagnostic and prognostic factors for LCS. Research has shown that several factors, including gender, the SARS-CoV-2 variant, pre-existing medical comorbidities, and Covid-19 vaccination, can influence the likelihood of developing LCS. Specifically, being female and having pre-existing medical comorbidities may increase the risk of experiencing long-term Covid-19 symptoms [11]. On the other hand, individuals who are fully vaccinated before SARS-CoV-2 infection and those infected with the Omicron variant (compared to other variants) may have a lower risk of developing LCS [12, 13]. The impact of older age and post-infection vaccination on the risk of LCS remains unclear [11, 12]. Moreover, potential targets for predicting and diagnosing LCS may involve the immune system’s cellular and molecular components, which could be linked to SARS-CoV-2 infection progression and subsequent chronic complications.

Due to their crucial role in stimulating adaptive immune responses, human leukocyte antigens (HLAs) are likely genetic indicators for predicting the prognosis of infectious diseases [14–16]. These molecules consist of a group of proteins expressed on the cell surface that stimulate cellular and humoral immune responses by presenting peptides derived from non-self-antigens to appropriate T cells [17]. HLA class I (HLA-I) molecules specifically present endogenous antigen-derived peptides, including viral antigens, to activate CD8^+^ T cells, while HLA class II (HLA-II) molecules present exogenous antigen-derived peptides to activate CD4^+^ T cells [18]. The major HLA-coding genes are located on the short arm of chromosome 6 (6q21) and are known as the most polymorphic region of the human genome [17]. The high degree of polymorphism and heterozygosity in the HLA genes enables the immune system to react dynamically to a broad range of pathogens and microorganisms, while also influencing the individuals’ susceptibility to infectious diseases [19]. Previous research has found a strong relationship between the specific HLA-I alleles carried by an individual and the severity of viral infections, as well as their long-term complications. For instance, individuals carrying the HLA-A*02 allele exhibit heightened protection against the human immunodeficiency virus (HIV), whereas those with the HLA-B*13 allele may be more susceptible to the progression and chronicity of HIV disease [20]. Furthermore, previous studies have found that individuals carrying the HLA-A*33 or -A*44 alleles are more susceptible to the progression of varicella-zoster virus (VZV) infection symptoms and to chronic and neurological symptoms. In contrast, individuals with the HLA-A*02 allele demonstrate effective control over the infection during its early stages [21, 22]. In the context of Covid-19, research has revealed potential associations between specific HLA-I alleles and variations in the severity and duration of the disease. Specifically, the HLA-B*35 allele has been associated with milder and more restricted Covid-19, while the HLA-A*11, -B*27, -B*51, and -C*01 alleles have been linked with severe Covid-19 [23–25].

The inability to promptly control infectious agents during the early stages of an infection, coupled with difficulties in completely clearing them during the later stages, may be the primary cause of severe and prolonged complications in individuals carrying certain HLA-I alleles [26, 27]. Repeated immune system activation due to the sustained presence of viral antigens, iatrogenic damage, and opportunistic secondary infections, can skew immune responses towards persistent and ineffective inflammation. This in turn can result in different long-term complications depending on the affected tissues and organs [28–30]. Recent studies have shown that high levels of inflammatory mediators, such as interleukin (IL)-1β, IL-6, tumor necrosis factor (TNF)-α, and C-reactive protein (CRP) after the acute phase of Covid-19, are associated with an increased incidence of neurological, pulmonary, cytoskeleton, and fatigue-related complications in LCS patients [31–33].

Therefore, investigating the interaction between the HLA-I alleles and inflammatory mediators in relation to the likelihood of LCS represent a highly practical approach to identify potential predictors of chronic inflammatory conditions and LCS following the acute phase of Covid-19. This endeavor can significantly contribute to the prevention and management of long-term complications, thereby reducing the healthcare burden and economic losses attributable to LCS. To achieve this objective, we conducted a comparative analysis of demographic characteristics, clinical history, serum levels of IL-6 and TNF-α, laboratory parameters, and the frequency of the HLA-I alleles in both LCS patients and their controls.

## Materials and methods

### Study design and participants

In this case–control study, all participants were selected among Covid-19-recovered individuals, who were referred to hospitals affiliated with the Isfahan University of Medical Sciences in Iran between July and September 2021 (the peak of the SARS-CoV-2 delta variant outbreak) due to acute Covid-19 and whose medical records contained all required details (i.e., details about the SARS-CoV-2 infection, Covid-19 severity during hospitalization, the duration of hospital and intensive care unit (ICU) stays, and any pre-existing medical conditions).

Participants had to meet the following eligibility criteria: Persian (Fars) ethnicity, aged between 30 and 65 years old, confirmation of a SARS-CoV-2 infection through reverse transcription-quantitative polymerase chain reaction (RT-qPCR) test and computerized tomography (CT) scan, classification of disease severity according to WHO’s interim guidance, and no pre-existing medical conditions (e.g., diabetes, hypertension, malignancy, immunodeficiency, autoimmune, or chronic inflammatory diseases). Furthermore, to minimize potential vaccine-related influences, we only included participants who had received two doses of the Sinopharm Covid-19 vaccine after recovery, with the last dose being at least a three months earlier. To prevent potential perturbation of inflammatory parameters, individuals who had experienced an infectious disease or received a vaccination within the past three months were excluded from the study. In addition, individuals with a history of smoking or drug addiction were also excluded.

**Fig. 1:**
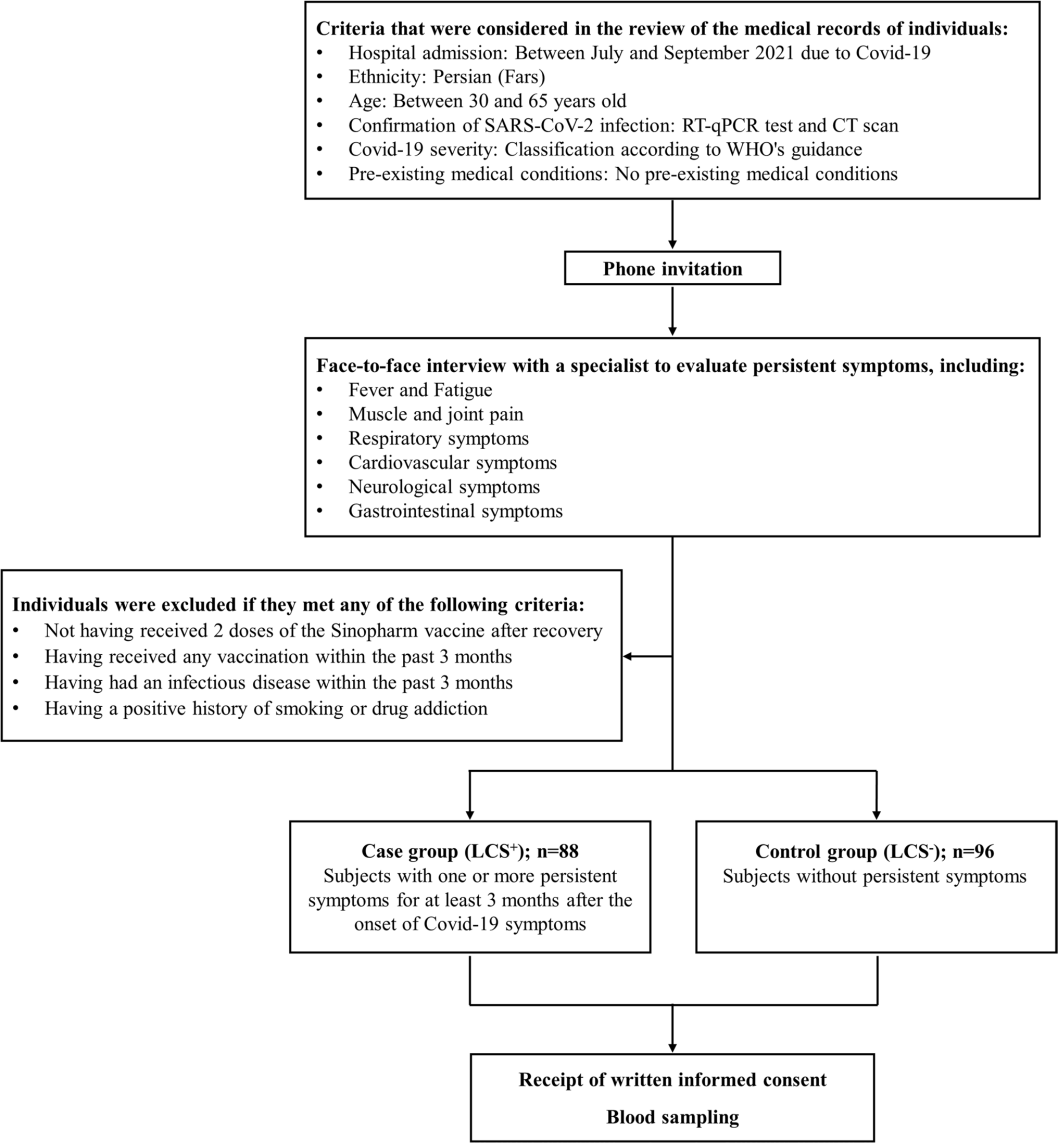
Flow diagram of participant selection. CT scan: computerized tomography scan, LCS: long Covid-19 syndrome, RT-qPCR: reverse transcription-quantitative polymerase chain reaction, SARS-CoV-2: severe acute respiratory syndrome coronavirus 2, WHO: world health organization

Eligible participants were contacted by phone and invited for a clinical examination and face-to-face interview with a specialist. During these interviews, a standardized questionnaire was used to investigate whether each participant had any persistent symptoms, including fever, headache and dizziness, tinnitus and earache, cough and shortness of breath, sore throat, fatigue, loss of smell and taste, cardiac problems, muscle and joint pain, insomnia, memory and concentration problems, depression and anxiety, gastrointestinal problems, hair loss, and vision problems. The symptoms were assessed using guidelines provided by the Centers for Disease Control and Prevention (CDC) and the National Health Service (NHS). Participants reporting one or more symptoms persisting from the onset of Covid-19 to at least three months later were classified into the LCS^+^ (case) group, while those without such symptoms were placed into the LCS^−^ (control) group. Following the receipt of written informed consent and the interview, 10 mL of whole blood was collected from each eligible participant for laboratory analysis. The flow diagram of participant selection is illustrated in Fig. 1.

This study adhered to the Declaration of Helsinki for research involving human participants and received ethical approval from the Ethics Committee of the Isfahan University of Medical Sciences, with the ethics code: IR.MUI.MED.REC.1400.795.

### Laboratory assessments

For the laboratory tests, 7 mL of blood from each participant was immediately sent to a clinical laboratory. The tests included complete blood count (CBC), assessment of prothrombin time (PT) and partial thromboplastin time (PTT), and measurement of various indicators, such as creatinine (Cr), alanine transaminase (ALT), aspartate aminotransferase (AST), lactate dehydrogenase (LDH), CRP, erythrocyte sedimentation rate (ESR), blood sugar (BS), and ferritin.

### Measurement of IL-6 and TNF-α

Serum was isolated from 3 mL of non-anticoagulated blood within 2 h. The serum concentrations of IL-6 and TNF-α were measured using a commercial Enzyme-Linked Immunosorbent Assay (ELISA) kit (Biolegend, USA) in accordance with the manufacturer’s instructions. The sensitivity of the ELISA kits was 4 pg/mL for IL-6 and 2 pg/mL for TNF-α.

### DNA extraction and HLA typing

Genomic DNA was extracted from 200 μL of the remaining blood samples using the proteinase K method and a DNA extraction kit (AddBio, Korea), in accordance with the manufacturer’s instructions. The quality and quantity of the extracted DNA were determined using NanoDrop (Thermo Scientific, UK). DNA samples with a concentration higher than 10 ng/μL were stored at −20 C prior to HLA testing.

HLA-A, -B, and -C allele typing was performed using the polymerase chain reaction with the sequence-specific primers (PCR-SSP) method, using the Olerup SSP® HLA-A-B-C SSP Combi Tray kit (Olerup, Sweden) in accordance with the manufacturer’s instructions. The PCR products were electrophoresed on a 2% agarose gel stained with DNA-safe stain, and specific bands were visualized under a UV transilluminator. HLA alleles were determined using both the software and worksheet methods provided by the manufacturer.

### Statistical analyses

Chi-square (χ^2^) and Fisher exact tests were used to compare the categorical variables between the LCS^−^ and LCS^+^ groups, and the results were presented as counts and percentages (%). For the quantitative variables, as the Kolmogorov–Smirnov test indicated the data was not normally distributed, differences between the groups were assessed using the Mann–Whitney U test. The results were reported as medians with interquartile ranges (IQR). Furthermore, to estimate the association between the variables and the likelihood of LCS, we performed a multiple logistic regression using the “Enter” method. We selected variables with a p-value ≤ 0.1 from the χ2 and Mann–Whitney U tests for inclusion in the logistic regression model. Due to its high correlation with white blood cell (WBC) and neutrophil-to-lymphocyte ratio (NLR), the lymphocyte count variable was excluded from the model. The odds ratios (OR) and corresponding 95% confidence intervals (95%CI) were calculated to measure the effect of the variables. All statistical analysis was performed using SPSS (v26.0), with a significance level of *P* < 0.05. GraphPad Prism (v9.0.0) software was used to create all figures.

## Results

### Demographic characteristics, laboratory parameters, and clinical history of the participants

We enrolled 88 subjects with LCS (43 males and 45 females) in the LCS^+^ group and 96 individuals without LCS (55 males and 41 females) in the LCS^−^ group. A comparison of demographic characteristics, laboratory parameters, and clinical histories of the two groups is presented in Table 1. There were no significant differences between the LCS^+^ and LCS^−^ groups for sex (*P* = 0.252) or age (*P* = 0.601). However, patients with LCS had a significantly higher body mass index (BMI) than their controls (*P* = 0.002).

**Table 1:** Comparison of the demographic characteristics, laboratory parameters, and clinical history of the two groups

Groups	LCS^+^ (*n* = 88)	LCS^−^ (*n* = 96)	*P*-value
Variable	**n (%) / Median (IQR1-IQR3)**	**n (%) / Median (IQR1-IQR3)**	
Sex			
Male	43 (43.90)	55 (56.10)	0.252
Female	45 (52.30)	41 (47.70)	
Age (years)	47.50 (39.00–55.25)	43.00 (37.25–58.00)	0.601
BMI (Kg/m^2^)	**28.48 (26.49–30.81)**	**25.95 (24.14–28.29)**	0.002*
WBC (× 10^3^µL)	**6.99 (5.95–7.97)**	**6.36 (5.80–7.36)**	0.016*
RBC (× 10^6^µL)	5.15 (4.84–5.50)	5.18 (4.88–5.63)	0.568
Hb (g/dL)	14.25 (13.50–15.70)	14.65 (13.42–15.67)	0.837
HCT (%)	43.55 (40.22–47.07)	44.85 (41.42–47.37)	0.171
Plt (× 10^3^µL)	319.00 (253.00–361.75)	290.50 (259.25–331.50)	0.129
Neu.C (× 10^3^µL)	4.00 (3.27–4.75)	3.72 (3.25–4.38)	0.208
Lym.C (× 10^3^µL)	**2.37 (1.95–3.02)**	**2.01 (1.79–2.48)**	< 0.001*
NLR	**1.60 (1.25–2.11)**	**1.77 (1.44–2.12)**	0.023*
ESR (mm/hr)	9.00 (4.00–13.00)	7.00 (4.00–11.00)	0.331
CRP (mg/L)	**2.40 (1.32–5.25)**	**1.80 (1.10–3.17)**	0.005*
LDH (U/L)	332.00 (291.00–425.00)	327.00 (291.00–387.00)	0.487
Ferritin (ng/mL)	**41.95 (26.26–82.58)**	**77.49 (32.57–114.16)**	0.012*
BS (mg/dL)	**97.50 (89.00–111.75)**	**92.50 (85.00–102.00)**	0.025*
Cr (mg/dL)	1.06 (0.91–1.19)	1.04 (0.90–1.17)	0.775
AST (U/L)	21.00 (19.00–24.00)	20.00 (18.00–24.00)	0.433
ALT (U/L)	20.50 (16.00–26.75)	22.00 (16.25–29.00)	0.285
PT (Sec)	13.00 (13.00–13.00)	13.00 (13.00–13.00)	0.706
PTT (Sec)	31.05 (29.22–33.00)	30.60 (29.37–32.77)	0.985
Duration of hospitalization in the acute phase	**8.50 (6.00–13.00)**	**0.00 (0.00–6.00)**	< 0.001*
ICU admission in the acute phase			
Yes	**21 (23.90)**	**4 (4.20)**	< 0.001*
No	**67 (76.10)**	**92 (95.80)**	
Covid-19 Severity in the acute phase			
Severe	**49 (55.70)**	**11 (11.50)**	< 0.001*
Non-Severe	**39 (44.30)**	**85 (88.50)**	

ALT: alanine transaminase, AST: aspartate transaminase, BMI: body mass index, BS: blood sugar, Cr: creatinine, CRP: c-reactive protein, dL: deciliter, ESR: erythrocyte sedimentation rate, g: gram, Hb: hemoglobin, HCT: hematocrit, hr: hour, ICU: intensive care unit, IQR: interquartile range, Kg: kilogram, L: liter, LCS: long Covid-19 syndrome, LDH: lactate dehydrogenase, Lym.C: lymphocyte count, m^2^: square meter, mg: milligram, mL: milliliter, mm: millimeter, n: number, Neu.C: neutrophil count, ng: nanogram, NLR: neutrophil/lymphocyte ratio, Plt: platelet, PT: prothrombin time, PTT: partial thromboplastin time, RBC: red blood cells, Sec: second, U: unit, WBC: white blood cells, µL: microliter. * indicates *P-value* < 0.05

The LCS^+^ group had significantly higher WBC, lymphocyte count, CRP, and BS, when compared to the LCS^−^ group (for all:* P* < 0.05). In contrast, the NLR and ferritin levels were significantly lower in the LCS^+^ group (*P* = 0.023 and *P* = 0.012; respectively). However, no significant differences were observed between the two groups in any of the other laboratory parameters (for all: *P* ≥ 0.05).

Moreover, when examining the clinical history of individuals during the acute phase of Covid-19, it became evident that subjects in the LCS^+^ group had significantly longer hospital stays, a higher rate of admission to ICU, and a higher incidence of severe Covid-19, in comparison to the LCS^−^ group (for all:* P* < 0.001).

### Clinical characteristics of patients with LCS

**Fig. 2:**
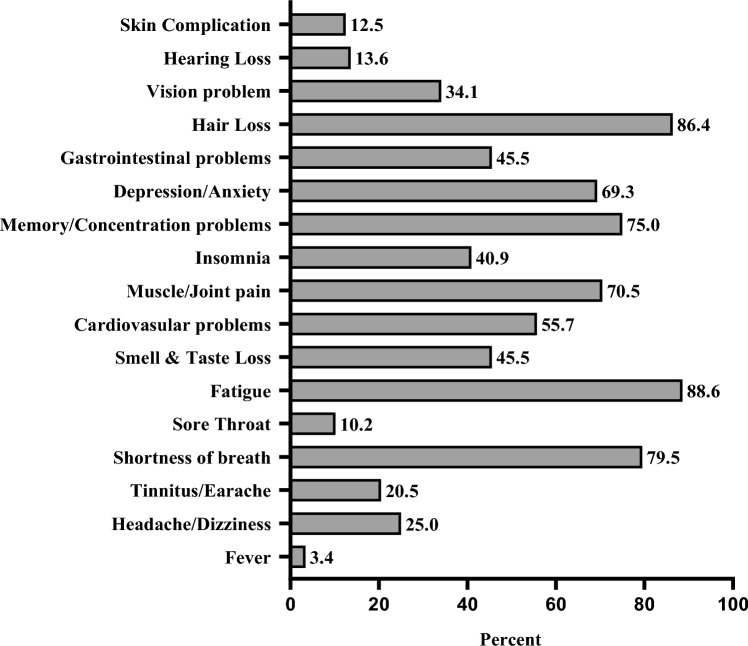
Symptom (lasting for > 12 weeks from the onset of Covid-19) prevalence in the LCS^+^ Group (%)

Figure 2 presents the prevalence of persistent symptoms in the LCS^+^ group. The most commonly reported persistent symptoms were fatigue (88.6%), hair loss (86.4%), shortness of breath (79.5%), memory/concentration problems (75.0%), and muscle/joint pain (70.5%). Moreover, the LCS^+^ participants who experienced severe Covid-19 during the acute phase exhibited a higher prevalence of enduring shortness of breath and memory/concentration problems, when compared to those with a history of non-severe Covid-19 (*P* = 0.008 and *P* = 0.009, respectively). However, please note that this data is not shown in the figure.

### The serum concentration of IL-6 and TNF-α

**Fig. 3:**
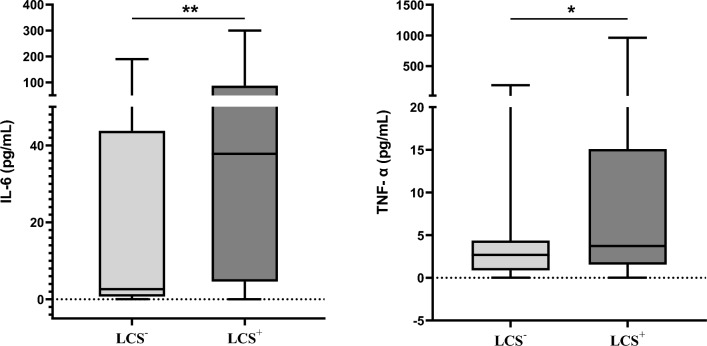
IL-6 and TNF-α serum levels in the LCS^+^ and LCS.^−^ groups. IL-6: interleukin-6, LCS: long Covid-19 syndrome, mL: milliliter, pg: picogram, TNF-α, tumor necrosis factor-α. * indicates *P-value* = 0.042, ** indicates *P-value* = 0.004

We also measured the serum levels of IL-6 and TNF-α in the two groups. This showed that the LCS^+^ group had higher serum levels of IL-6 than the LCS^−^ group (37.78 (4.60–87.33) vs. 2.62 (0.72–43.76)), and this difference was statistically significant (*P* = 0.004; Fig. 3). Similarly, the serum level of TNF-α was significantly higher in the LCS^+^ group (3.73 (1.55–15.09)) than in the LCS^−^ group (2.68 (0.87–4.36), *P* = 0.042; Fig. 3).

### The frequency of HLA-A, -B, and -C alleles

The frequencies of the HLA-A, -B, and -C alleles in the LCS^+^ and LCS^−^ groups are presented in Table 2. The HLA-A*11, -B*14, -B*38, -B*50, and -C*07 alleles were all more frequently present in the LCS^+^ group than in the LCS^−^ group (for all: *P* < 0.05). In contrast, the HLA-A*32, -B*51, -C*08, and -C*12 alleles were less frequently found in the LCS^+^ group (for all: *P* < 0.05).

**Table 2:** The frequencies of the HLA-A, -B, and -C alleles in the LCS^+^ and LCS^−^ groups

Alleles	LCS^+^ n (%)	LCS^−^ n (%)	P-value	OR (95%CI)	Alleles	LCS^+^ n (%)	LCS^−^ n (%)	P-value	OR (CI95%)	Alleles	LCS^+^ n (%)	LCS^−^ n (%)	P-value	OR (CI95%)
A*01	21 (11.9)	20 (10.4)	0.644	1.16 (0.60–2.23)	B*07	10 (5.7)	8 (4.2)	0.501	1.38 (0.53–3.59)	C*01	6 (3.4)	4 (2.1)	0.529	1.65 (0.46–5.97)
A*02	22 (12.5)	24 (12.5)	1.000	1.00 (0.53–1.85)	B*08	4 (2.3)	3 (1.6)	0.714	1.46 (0.32–6.64)	C*02	12 (6.8)	15 (7.8)	0.715	0.86 (0.39–1.89)
A*03	18 (10.2)	30 (15.6)	0.125	0.61 (0.33–1.14)	B*13	8 (4.5)	7 (3.6)	0.663	1.25 (0.44–3.54)	C*03	4 (2.3)	4 (2.1)	1.000	1.09 (0.26–4.43)
A*11	**29 (16.5)**	**17 (8.9)**	0.027*	**2.03 (1.07–3.84)**	B*14	**12 (6.8)**	**1 (0.5)**	0.001*	**13.97 (1.79–108.63)**	C*04	26 (14.8)	26 (13.5)	0.735	1.10 (0.61–1.99)
A*23	4 (2.3)	7 (3.6)	0.440	0.61 (0.17–2.13)	B*15	5 (2.8)	4 (9.7)	0.355	0.59 (0.19–1.80)	C*05	22 (12.5)	17 (8.9)	0.256	1.47 (0.75–2.87)
A*24	37 (21.0)	44 (22.9)	0.661	0.89 (0.54–1.46)	B*18	8 (4.5)	14 (7.3)	0.267	0.60 (0.24–1.48)	C*06	23 (13.1)	18 (9.4)	0.261	1.45 (0.75–2.79)
A*25	NS	1 (0.5)	1.000	NA	B*35	29 (16.5)	43 (22.4)	0.153	0.68 (0.40–1.15)	C*07	**31 (17.6)**	**17 (8.9)**	0.013*	**2.20 (1.17–4.13)**
A*26	12 (6.8)	10 (5.2)	0.515	1.33 (0.56–3.16)	B*37	2 (1.1)	3 (1.6)	1.000	0.72 (0.12–4.38)	C*08	**7 (4.0)**	**32 (16.7)**	< 0.001*	**0.20 (0.08–0.48)**
A*29	4 (2.3)	2 (1.0)	0.432	2.20 (0.40–12.21)	B*38	**12 (6.8)**	**2 (1.0)**	0.004*	**6.95 (1.53–31.51)**	C*12	**12 (6.8)**	**25 (13.0)**	0.048*	**0.48 (0.23–1.00)**
A*30	7 (4.0)	8 (4.2)	0.927	0.95 (0.33–2.68)	B*39	2 (1.1)	3 (1.6)	1.000	0.72 (0.12–4.38)	C*14	9 (5.1)	7 (3.6)	0.490	1.42 (0.51–3.90)
A*31	2 (1.1)	1 (0.5)	0.608	2.19 (0.19–24.42)	B*40	8 (4.5)	9 (4.7)	0.948	0.96 (0.36–2.56)	C*15	3 (1.7)	6 (3.1)	0.506	0.53 (0.13–2.18)
A*32	**4 (2.3)**	**14 (7.3)**	0.026*	**0.29 (0.09–0.91)**	B*41	4 (2.3)	6 (3.1)	0.753	0.72 (0.20–2.59)	C*16	14 (8.0)	20 (10.4)	0.415	0.74 (0.36–1.52)
A*33	10 (5.7)	6 (3.1)	0.230	1.86 (0.66–5.24)	B*44	12 (6.8)	12 (6.3)	0.825	1.09 (0.48–2.51)	C*17	3 (1.7)	NS	0.108	NA
A*66	NS	1 (0.5)	1.000	NA	B*47	2 (1.1)	NS	0.228	NA	C*18	4 (2.3)	1 (0.5)	0.198	4.44 (0.49–40.12)
A*68	5 (2.8)	6 (3.1)	0.873	0.90 (0.27–3.02)	B*48	1 (0.6)	2 (1.0)	1.000	0.54 (0.04–6.03)					
A*69	1 (0.6)	1 (0.5)	1.000	1.09 (0.06–17.58)	B*49	9 (5.1)	9 (4.7)	0.850	1.09 (0.42–2.82)					
					B*50	**9 (5.1)**	**2 (1.0)**	0.022*	**5.12 (1.09–24.03)**					
					B*51	**14 (8.0)**	**32 (16.7)**	0.012*	**0.43 (0.22–0.84)**					
					B*52	7 (4.0)	7 (3.6)	0.868	1.09 (0.37–3.18)					
					B*53	1 (0.6)	4 (2.1)	0.374	0.26 (0.03–2.42)					
					B*55	6 (3.4)	6 (3.1)	0.878	1.09 (0.34–3.45)					
					B*56	1 (0.6)	1 (0.5)	1.000	1.09 (0.06–17.58)					
					B*57	4 (2.3)	2 (1.0)	0.432	2.20 (0.40–12.21)					
					B*58	5 (2.8)	1 (0.5)	0.108	5.58 (0.64–48.27)					
					B*73	1 (0.6)	4 (2.1)	0.374	0.26 (0.03–2.42)					

LCS: long Covid-19 syndrome, n: number, NA: not applicable, NS: not seen, OR: odds ratio, 95%CI: 95% confidence interval. * indicates *P-value* < 0.05

Furthermore, the frequency of the alleles differed in accordance with the severity of Covid-19 during the acute phase of the disease. Specifically, we found the HLA-A*11, -B*08, -B*14, B*50, and -C*07 alleles were significantly more common in patients who experienced severe symptoms during the acute phase of the disease (SC group), when compared to those with a history of non-severe Covid-19 (NSC group) (for all,* P* < 0.05). In contrast, the HLA-A*32, -B*35, -B*51, and -C*08 alleles were less frequently found in the SC group than in the NSC group (for all,* P* < 0.05; Supplementary Table 1).

### Multiple logistic regression modeling for screening LCS

The results from the multiple logistic regression model are shown in Table 3. After adjusting for the most important variables, including BMI, WBC, NLR, CRP, ferritin, BS, IL-6, TNF-α, HLA-A*11,-A*32, -B*14, -B*38, -B*50, -B*51, -C*07, -C*08, -C*12 and severe Covid-19, we found that the likelihood of experiencing LCS was significantly associated with BMI, CRP, ferritin, IL-6, the HLA-A*11, -C*07, and -C*08 alleles, as well as a positive history of severe Covid-19. These results indicate that there is a substantially higher likelihood of LCS in individuals who experienced severe symptoms during the acute phase of Covid-19, in comparison to those with a history of non-severe Covid-19 [OR: 4.75, 95% CI: 1.56–14.44]. Similarly, both the HLA-A*11 and -C*07 alleles were linked to an increased likelihood of LCS [OR: 2.32, 95% CI: 1.13–4.74, and OR: 2.16, 95% CI: 1.08–4.35, respectively]. In contrast, the presence of the HLA-C*08 allele had a significant protective effect against LCS [OR: 0.06, 95% CI: 0.01–0.60]. Moreover, the odds of LCS were significantly increased with each one-unit rise in BMI (kg/m^2^) [OR: 1.12, 95% CI: 1.00–1.25], CRP (mg/L) [OR: 1.21, 95% CI: 1.05–1.38], or serum IL-6 (pg/mL) [OR: 1.01, 95% CI: 1.00–1.01]. Conversely, each one-unit rise in ferritin (ng/mL) was associated with a significant decrease in the odds of LSC [(OR: 0.99, 95% CI: 0.98–1.00].

**Table 3:** The results of the multiple logistic regression showing the associations between the variables and the odds of LCS

Variable	B	S.E	Wald	df	*P*-value	OR	95% C.I. for OR	
							Lower	Upper
**BMI**	**0.114**	**0.057**	**3.994**	**1**	0.047*	**1.12**	**1.00**	**1.25**
WBC	0.373	0.284	1.734	1	0.188	1.45	0.83	2.53
NLR	-0.309	0.533	0.337	1	0.561	0.73	0.26	2.09
**CRP**	**0.186**	**0.070**	**7.141**	**1**	0.008*	**1.21**	**1.05**	**1.38**
**Ferritin**	**-0.013**	**0.006**	**3.991**	**1**	0.046*	**0.99**	**0.98**	**1.00**
BS	0.035	0.019	3.287	1	0.070	1.04	0.99	1.08
**IL-6**	**0.007**	**0.004**	**4.300**	**1**	0.038*	**1.01**	**1.00**	**1.01**
TNF-α	0.003	0.003	1.104	1	0.293	1.00	0.99	1.01
A*11	**0.840**	**0.365**	**5.299**	**1**	0.021*	**2.32**	**1.13**	**4.74**
A*32	-1.137	1.069	1.131	1	0.288	0.32	0.04	2.61
B*14	1.618	1.322	1.498	1	0.221	5.04	0.38	67.32
B*38	0.945	1.347	0.492	1	0.483	2.57	0.18	36.03
B*50	-1.772	2.063	0.738	1	0.390	0.17	0.01	9.70
B*51	0.041	0.704	0.003	1	0.954	1.04	0.26	4.14
C*07	**0.772**	**0.357**	**4.675**	**1**	0.031*	**2.16**	**1.08**	**4.35**
C*08	**-2.842**	**1.190**	**5.704**	**1**	0.017*	**0.06**	**0.01**	**0.60**
C*12	-0.743	0.392	3.593	1	0.058	0.48	0.22	1.03
**Severe Covid-19**	**1.557**	**0.568**	**7.525**	**1**	0.006*	**4.75**	**1.56**	**14.44**

B: regression coefficient, BMI: body mass index, BS: blood sugar, CRP: c-reactive protein, df: degrees of freedom, IL-6: interleukin-6, LCS: long Covid-19 syndrome, NLR: neutrophil/lymphocyte ratio, OR: odds ratio, S.E.: standard error, TNF-α: tumor necrosis factor-α, WBC: white blood cell, 95%CI: 95% confidence interval. * indicates *P-value* < 0.05

## Discussion

The present study investigated differences in the likelihood of experiencing LCS using demographic characteristics, laboratory indicators, clinical history, and the frequency of the HLA-I alleles. The results showed that the odds of LCS increase by 0.12 times per one-unit rise in BMI. Previous findings have shown that individuals with a BMI greater than 30 kg/m^2^ have a 10% higher relative risk of experiencing long-term Covid-19 complications than those with a normal BMI [34]. Furthermore, obesity has been identified as an independent risk factor for an incomplete recovery, even one year after hospital discharge, among patients who have recovered from severe or critical Covid-19 [35]. The underlying mechanism for this phenomenon has been attributed to mitochondrial dysfunction and the production of reactive oxygen species caused by hyperglycemia in individuals with obesity. These processes lead to the production of pro-inflammatory mediators like leptin (a pro-inflammatory adipokine), TNF-α, IL-1, IL-6, and IL-18 [36]. These mediators can skew the immune response towards a low-grade chronic local inflammatory state, resulting in tissue damage, necrosis, and cellular dysfunction. Over time, both innate and adaptive immune responses are systemically affected, rendering obese individuals more vulnerable to viral persistence and secondary infections [36, 37].

Additionally, our study found a strong association between the severity of Covid-19 during the acute phase of the disease and the likelihood of LCS. Specifically, individuals who experienced severe symptoms during the acute stage of Covid-19 had a 3.75 times higher likelihood of developing LCS, in comparison to those with a history of non-severe Covid-19. Previous studies have reported that patients who experienced severe Covid-19 and required invasive mechanical ventilation, ICU admission, and prolonged hospital stays were more likely to develop long-term tissue damage and persistent symptoms [38–40]. This may be due to the systemic inflammatory responses and cytokine storms induced by severe Covid-19. The excessive production of pro-inflammatory cytokines can exacerbate tissue damage by stimulating the cytotoxic activity of the CD8^+^ T cells, promoting apoptosis, and disrupting the control of leukocyte trafficking [41, 42]. In particular, IL-6 fosters the differentiation of naïve CD4^+^ T cells into Th17 cells and inhibits Treg differentiation, resulting in immunological tolerance disruption and the development of chronic inflammation [43]. TNF-α further exacerbates this process by inducing IL-6 expression [44]. Moreover, some required medical procedures for patients with severe Covid-19, such as intubation, sometimes cause chronic inflammation due to iatrogenic damage. In addition, the overuse of corticosteroids in patients with severe Covid-19 can result in the development of opportunistic secondary infections, triggering sustained inflammatory responses [45]. The sustained inflammatory condition observed in LCS may also be due to the persistence of SARS-CoV-2 antigens in tissues, autoreactive reactions, and the failure of the individual’s immune system to return to baseline homeostasis, which may underlie the development of long-term Covid-19 symptoms [32].

Among the long-term symptoms, in our study we found that fatigue, hair loss, shortness of breath, and concentration and memory problems were the most commonly reported by individuals with LCS, which is consistent with previous findings [46–48]. Previous research has shown that the production of pro-inflammatory cytokines such as IL-1β, IL-6, IL-12, interferon (IFN)-γ, and TNF-α leads to the recruitment and activation of Th1 and Th17 cells on microglia, resulting in nerve fiber demyelination, which may explain the chronic muscle fatigue and neurological disorders in individuals with LCS [33, 49, 50]. In addition, persistent inflammation, lung fibrosis, and damage to pulmonary vasculature caused by pro-inflammatory cytokines such as IL-6 may potentially contribute to long-term respiratory symptoms [1, 51]. In line with these findings, our study showed that higher levels of CRP and IL-6 were associated with LCS. Specifically, we found that the odds of LCS increased by 0.21 and 0.01-fold for each one-unit rise in CRP and IL-6, respectively. In contrast, higher levels of ferritin were associated with lower odds of LCS (OR = 0.99). It is worth noting that previous studies have reported contradictory findings regarding ferritin, which could be attributed to the timing of marker evaluations in relation to the acute phase of the disease. Nevertheless, understanding this issue highlights the need for further studies [32, 52–54].

The findings of a study have highlighted the vital role of HLA molecules in the immune system, since they act as crucial mediators in the defense against infections and significantly impacting the development and persistence of the clinical symptoms [55]. As the most polymorphic molecules, HLA-I play a crucial role in mitigating clinical complications by selectively presenting a wide array of viral antigen-derived peptides to the CD8^+^ T cells, which are central cells of the acquired immune system’s defense against viral infections [56]. In the context of the SARS-CoV-2 infection, previous studies have shown the HLA-I alleles to be associated with variations in Covid-19 severity, as well as the resulting outcomes. Furthermore, several studies have found susceptibility to Covid-19 to be associated with the HLA-B*08 [57], -B*35 [58], and -C*07 [59] alleles. In addition, individuals carrying the HLA-A*11 and -B*51 alleles have a heightened susceptibility to severe Covid-19 and worse outcomes in comparison to those lacking these alleles [60]. Moreover, patients who carry the HLA-A*11 allele have been observed to have higher mortality rates from Covid-19 [61]. In contrast, the HLA-A*32 [24, 58] and -B*14 [57] alleles have been found to have a protective effect against Covid-19. However, to the best of our knowledge, no previous research has investigated the relationship the HLA-I alleles have with LCS.

The current research found significantly higher frequencies of the HLA-A*11, -B*14, -B*38, -B*50, and -C*07 alleles in the LCS group than in the control group, while lower frequencies of HLA-A*32, -B*51, and -C*08 alleles were observed in the LCS group. After adjusting for the important variables, the HLA-A*11 and -C*07 alleles were still associated with an increased likelihood of LCS (1.32 and 1.16-fold, respectively). In contrast, the HLA-C*08 allele was found to have a protective effect against LCS (OR = 0.06). Considering the higher frequencies of the HLA-A*11 and -C*07 alleles in patients who experienced severe symptoms during the acute phase of the disease, compared to those with a history of non-severe Covid-19, we posit a hypothetical mechanism to explain the occurrence of long Covid-19 syndrome. This mechanism involves the incomplete clearance of SARS-CoV-2 during the acute phase of the disease in individuals carrying these alleles, ultimately leading to tissue damage due to persistent and inefficient inflammatory responses over the long term [2].

It is reasonable to assume that the HLA-I alleles with a stronger binding affinity for epitopes derived from the SARS-CoV-2 antigens elicit more effective CTL responses. Consequently, this prevents disease progression to severe complications, facilitates complete virus clearance, and promotes full recovery. Conversely, HLA-I alleles with a weaker binding affinity are associated with sustained inflammation, organ damage, and long-term complications. This is because they trigger inefficient and dysregulated immune responses that are unable to clear SARS-CoV-2 [55, 62]. In this context, Meysman et al. previously reported that HLA-A*02:01-restricted peptides triggered more robust immune responses compared to HLA-A*24:02-restricted peptides, when examining VZV IE-62-derived peptides [63]. For SARS-CoV-2 infection, the HLA-A*01:01, -A*02:01, -A*24:02, -B*15:01, and -B*44:02-restricted peptides were shown to efficiently induce epitope-specific CD8^+^ T-cell responses [64], while no specific spike-derived peptides were identified for the HLA-B*08:01 and -B*27:05 alleles, suggesting the absence of appropriate spike-specific CD8^+^ T cell responses in individuals carrying these alleles [65].

## Limitations and future directions

It is important to acknowledge the limitations of our study, which should be considered in future research. One limitation was the absence of well-defined diagnostic criteria for LCS, potentially impacting the accuracy of classifying individuals into the LCS and non-LCS groups. In addition, the small sample size posed challenges and may have influenced the results. The scarcity of similar studies further restricts our ability to make comparisons. Furthermore, our study followed a case–control design and only assessed participants at a single point in time. Therefore, we recommend conducting larger-scale longitudinal studies in diverse ethnic populations to investigate changes in symptoms and levels of inflammatory markers in recovered Covid-19 patients over time.

## Conclusion

Our study showed that an increased likelihood of LCS was associated with higher BMI, a history of severe Covid-19 during the acute phase of the disease, the presence of the HLA-A*11 and -C*07 alleles, as well as elevated levels of serum CRP and IL-6. In contrast, the HLA-C*08 allele and high serum ferritin levels significantly decreased the odds of LCS.

## Supplementary Information

Below is the link to the electronic supplementary material.

Supplementary file1 (DOCX 21 KB)

## Data Availability

Not applicable.
