# Supplementary material for: The demographic, laboratory and genetic factors associated with long Covid-19 syndrome: a case–control study
Source: Clin Exp Med. 2024 Jan 17;24(1):1. doi: 10.1007/s10238-023-01256-1 (PMC10794331; doi:10.1007/s10238-023-01256-1)
Supplement: Supplementary file 1 — Supplementary file1 (DOCX 21 KB) [file 10238_2023_1256_MOESM1_ESM.docx]

**Supplementary Table 1** The frequencies of HLA-A, -B, and -C alleles in SC and NSC groups

| Alleles | SC  n (%) | | NSC  n (%) | | P-value | OR (95%CI) | Alleles | SC  n (%) | NSC  n (%) | P-value | OR (95%CI) | Alleles | SC  n (%) | NSC  n (%) | P-value | OR (95%CI) |
| --- | --- | --- | --- | --- | --- | --- | --- | --- | --- | --- | --- | --- | --- | --- | --- | --- |
| A*01 | 14 (11.7) | | 27 (10.9) | | 0.824 | 1.08 (0.54-2.14) | B*07 | 6 (5.0) | 12 (4.8) | 0.946 | 1.03 (0.37-2.82) | C*01 | 3 (2.5) | 7 (2.8) | 1.000 | 0.88 (0.22-3.47) |
| A*02 | 13 (10.8) | | 33 (13.3) | | 0.501 | 0.79 (0.40-1.56) | **B*08** | **5 (4.2)** | **2 (0.8)** | **0.040*** | **5.34 (1.02-27.97)** | C*02 | 10 (8.3) | 17 (6.9) | 0.610 | 1.23 (0.54-2.78) |
| A*03 | 13 (10.8) | | 35 (14.1) | | 0.381 | 0.73 (0.37-1.45) | B*13 | 5 (4.2) | 10 (4.0) | 0.951 | 1.03 (0.34-3.09) | C*03 | 3 (2.5) | 5 (2.0) | 1.000 | 1.24 (0.29-5.30) |
| **A*11** | **24 (20.0)** | | **22 (8.9)** | | **0.002*** | **2.56 (1.37-4.80)** | **B*14** | **9 (7.5)** | **4 (1.6)** | **0.007*** | **4.94 (1.49-16.40)** | C*04 | 20 (16.7) | 32 (12.9) | 0.331 | 1.35 (0.73-2.47) |
| A*23 | 4 (3.3) | | 7 (2.8) | | 0.787 | 1.18 (0.34-4.13) | B*15 | 4 (3.3) | 10 (4.0) | 0.784 | 0.82 (0.25-2.67) | C*05 | 18 (15.0) | 21 (8.5) | 0.056 | 1.90 (0.97-3.73) |
| A*24 | 24 (20.0) | | 57 (23.0) | | 0.517 | 0.83 (0.49-1.43) | B*18 | 4 (3.3) | 18 (7.3) | 0.137 | 0.44 (0.14-1.33) | C*06 | 10 (8.3) | 31 (12.5) | 0.234 | 0.63 (0.30-1.34) |
| A*25 | NS | | 1 (0.4) | | 1.000 | NA | **B*35** | **16 (13.3)** | **56 (22.6)** | **0.036*** | **0.52 (0.28-0.96)** | **C*07** | **25 (20.8)** | **23 (9.3)** | **0.002*** | **2.57 (1.39-4.76)** |
| A*26 | 7 (5.8) | | 15 (6.0) | | 0.935 | 0.96 (0.38-2.42) | B*37 | 1 (0.8) | 4 (1.6) | 0.672 | 0.51 (0.05-4.63) | **C*08** | **2 (1.7)** | **37 (14.9)** | **<0.001*** | **0.09 (0.02-0.40)** |
| A*29 | 4 (3.3) | | 2 (0.8) | | 0.091 | 4.24 (0.76-23.49) | B*38 | 6 (5.0) | 8 (3.2) | 0.562 | 1.57 (0.53-4.65) | C*12 | 9 (7.5) | 28 (11.3) | 0.257 | 0.63 (0.29-1.39) |
| A*30 | 3 (2.5) | | 12 (4.8) | | 0.403 | 0.50 (0.14-1.82) | B*39 | 2 (1.7) | 3 (1.2) | 1.000 | 1.38 (0.22-8.39) | C*14 | 5 (4.2) | 11 (4.4) | 0.906 | 0.93 (0.31-2.75) |
| A*31 | 1 (0.8) | | 2 (0.8) | | 1.000 | 1.03 (0.09-11.51) | B*40 | 5 (4.2) | 12 (4.8) | 0.773 | 0.85 (0.29-2.48) | C*15 | 3 (2.5) | 6 (2.4) | 1.000 | 1.03 (0.25-4.20) |
| **A*32** | **2 (1.7)** | | **16 (6.5)** | | **0.046*** | **0.24 (0.05-1.08)** | B*41 | 2 (1.7) | 8 (3.2) | 0.509 | 0.50 (0.10-2.43) | C*16 | 11 (9.2) | 23 (9.3) | 0.973 | 0.98 (0.46-2.09) |
| A*33 | 8 (3.2) | | 8 (3.2) | | 0.129 | 2.14 (0.78-5.85) | B*44 | 10 (8.3) | 14 (5.6) | 0.328 | 1.51 (0.65-3.52) | C*17 | NS | 3 (1.2) | 0.554 | NA |
| A*66 | NS | | 1 (0.4) | | 1.000 | NA | B*47 | 2 (1.7) | NS | 0.106 | NA | C*18 | 1 (0.8) | 4 (1.6) | 0.672 | 0.51 (0.05-4.63) |
| A*68 | 3 (2.5) | | 8 (3.2) | | 0.761 | 0.76 (0.20-2.95) | B*48 | 2 (1.7) | 1 (0.4) | 0.206 | 4.18 (0.37-46.63) |  |  |  |  |  |
| A*69 | NS | | 2 (0.8) | | 0.559 | NA | B*49 | 7 (5.8) | 11 (4.4) | 0.560 | 1.33 (0.50-3.53) |  |  |  |  |  |
|  |  |  | |  | |  | **B*50** | **7 (5.8)** | **4 (1.6)** | **0.044*** | **3.77 (1.08-13.17)** |  |  |  |  |  |
|  |  |  | |  | |  | **B*51** | **9 (7.5)** | **37 (14.9)** | **0.044*** | **0.46 (0.21-0.99)** |  |  |  |  |  |
|  |  |  | |  | |  | B*52 | 7 (5.8) | 7 (2.8) | 0.243 | 2.13 (0.73-6.22) |  |  |  |  |  |
|  |  |  | |  | |  | B*53 | NS | 5 (2.0) | 0.178 | NA |  |  |  |  |  |
|  |  |  | |  | |  | B*55 | 5 (4.2) | 7 (2.8) | 0.537 | 1.49 (0.46-4.81) |  |  |  |  |  |
|  |  |  | |  | |  | B*56 | NS | 2 (0.8) | 0.559 | NA |  |  |  |  |  |
|  |  |  | |  | |  | B*57 | 2 (1.7) | 4 (1.6) | 1.000 | 1.03 (0.18-5.72) |  |  |  |  |  |
|  |  |  | |  | |  | B*58 | 4 (3.3) | 2 (0.8) | 0.091 | 4.24 (0.76-23.49) |  |  |  |  |  |
|  |  |  | |  | |  | B*73 | NS | 5 (2.0) | 0.117 | NA |  |  |  |  |  |

n: number, NA: not applicable, NS: not seen, NSC: non-severe Covid-19, SC: severe Covid-19, OR: odds ratio, 95%CI: 95% confidence interval. *** indicates *P-value*<0.05**
